# Integration of single-cell and bulk RNA-seq to establish a predictive signature based on the differentiation trajectory of M2 macrophages in lung adenocarcinoma

**DOI:** 10.3389/fgene.2022.1010440

**Published:** 2022-09-12

**Authors:** Zhike Chen, Jian Yang, Yu Li, Weibiao Zeng, Yiling Bai, Cheng Ding, Chun Xu, Chang Li, Jun Chen, Sheng Ju, Lijuan Tang, Jun Zhao

**Affiliations:** ^1^ Department of Thoracic Surgery, The First Affiliated Hospital of Soochow University, Suzhou, China; ^2^ Institute of Thoracic Surgery, The First Affiliated Hospital of Soochow University, Suzhou, China; ^3^ Department of Oncology, The First Affiliated Hospital of Soochow University, Suzhou, China; ^4^ Dalian Medical University, Dalian, Liaoning, China; ^5^ Department of Pathology, Affiliated Hospital of Nantong University, Nantong, Jiangsu, China

**Keywords:** lung adenocarcinoma, tumor microenvironment (TME), M2 macrophages, ScRNA-seq, immunotherapy

## Abstract

**Background:** Tumor-associated macrophages as important members of the tumor microenvironment, are highly plastic and heterogeneous. TAMs can be classified into two preliminary subtypes: M1 and M2 macrophages. M2 macrophages are significantly associated with the progression of lung adenocarcinoma. However, no study has investigated the heterogeneity among M2 macrophages and their differentiation-related genes at the single-cell level to guide the clinical treatment of lung adenocarcinoma.

**Methods:** Using the available annotation information from the Tumor Immune Single-cell Hub database, we clustered and annotated 12 lung adenocarcinoma samples using the R package ‘Seurat’. Subsequently, we extracted M2 macrophages for secondary clustering analysis and performed cell trajectory analysis using the R package ‘monocle2’. Based on heterogeneous genes associated with the differentiation trajectory of M2 macrophages, we established a prognostic lung adenocarcinoma model using Lasso-Cox and multivariate stepwise regression. In addition, we also performed immunotherapy and chemotherapy predictions.

**Results:** M2 macrophages exhibit heterogeneity among themselves. M2 macrophages in different differentiation states showed significant differences in pathway activation and immune cell communication. Prognostic signature based on heterogeneous genes can be used to classify the prognostic status and abundance of immune cell infiltration in lung adenocarcinoma patients. In addition, the calculation of the Tumor Immune Dysfunction and Exclusion (TIDE) algorithm and the validation of the GSE126044 database indicated that lung adenocarcinoma patients with high-risk scores had poorer treatment outcomes when receiving immune checkpoint inhibitors treatment.

**Conclusion:** Based on scRNA-seq and Bulk-seq data, we identified M2 macrophage-associated prognostic signature with a potential clinical utility to improve precision therapy.

## Introduction

Lung cancer is one of the most common malignancies and the leading cause of cancer-related deaths. There are approximately two million new cases and 1.76 million deaths each year (Thai et al., 2021). It can be divided into two types: non-small cell lung cancer (NSCLC) and small cell lung cancer (SCLC) (Thai et al., 2021). NSCLC is the leading type of lung cancer, accounting for about 85% of the total lung cancers (Srivastava et al., 2022). Meanwhile, Lung adenocarcinoma is the most common histological subtype of NSCLC. Lung adenocarcinoma has a strong heterogeneity and a complex tumor microenvironment (TME) (He et al., 2021). Traditional pathological stages do not fully determine the prognosis of NSCLC patients. Therefore, the development of novel and reliable prognostic models can help stratify the risk of lung adenocarcinoma patients and provide targeted immunotherapy and chemotherapy strategies (Shi et al., 2021).

TME, an ecosystem with a complex communication network, consists of tumor cells, cancer-associated stromal and immune cells, and other non-cellular components (Wu and Dai, 2017; Maacha et al., 2019). Numerous studies have shown that the development, progression, and metastasis of lung adenocarcinoma are closely related to TME (Kamata et al., 2020; Kim et al., 2020; Li et al., 2021). Macrophages are monocyte-derived immune cells with many biological functions and are also essential members of the TME (Varol et al., 2015). Tumor-associated macrophages are functionally heterogeneous and can be classified into two subtypes: M1 macrophages and M2 macrophages (Yunna et al., 2020). M1 macrophages inhibit angiogenesis and tumor progression. A growing body of literature has reported that, unlike M1 macrophages, M2 macrophages significantly promote angiogenesis, metastasis, and tumor growth (Guo et al., 2019; Zhang et al., 2019; Pan et al., 2020). Furthermore, crosstalk between M2 macrophages and immune cells (or molecules) can also promote tumor escape. Therefore, M2 macrophages are key components in the development of the tumor immunosuppressive microenvironment (Zhou et al., 2020) and would be of great scientific value to investigate the effects of M2 macrophages on lung adenocarcinoma patients (Pan et al., 2020). Traditional transcriptome sequencing techniques lose information on heterogeneity between cells as all cells in a tumor sample are treated as a whole. Thus, single-cell sequencing is a good way to characterize heterogeneity between cells (Wu F. et al., 2021). Exploring the key genes that determine cell heterogeneity in the differentiation trajectory of M2 macrophages using single-cell sequencing might help determine the prognosis of lung adenocarcinoma patients and provide valuable guidance for clinical strategies.

In this study, we first uncovered genes associated with the heterogeneity of M2 macrophages based on single-cell sequencing data. Next, we performed a univariate Cox analysis of all heterogeneous genes and extracted prognostic genes which might be relevant to lung adenocarcinoma development and progression. Based on prognostic-related genes, we performed a Lasso-Cox and multivariate stepwise regression analysis and constructed a prognostic model for lung adenocarcinoma patients (Long et al., 2021). In this model, the risk score was an independent prognostic factor for lung adenocarcinoma patients and had a higher prognostic accuracy than clinical factors. After combining the clinical factors, we constructed a nomogram for a more accurate prognostic evaluation. Together, our results showed that heterogeneous genes associated with the differentiation of M2 macrophages uncovered from single-cell sequencing data could characterize the prognostic status of lung adenocarcinoma patients. The prognostic signature we established has clinical potential to predict the efficacy of immunotherapy (ICIs) and chemotherapy.

## Materials and methods

### Data collection

Twelve single-cell RNA sequencing samples from five lung adenocarcinoma patients in the GSE127465 database were included in this study. Bulk sequencing data, mutation data, and clinical information for lung adenocarcinoma patients were downloaded from The *Cancer* Genome Atlas (TCGA, https://portal.gdc.cancer.gov/) database. Microarray sequencing data and clinical information of the GSE31210 database were downloaded from Gene Expression Omnibus (GEO, https://www.ncbi.nlm.nih.gov/geo/) as an external independent validation set for the prognostic signature. Furthermore, the GSE126044 database was used as the immunotherapy response validation cohort (anti-PD-1 treatment). Detailed clinical information for TCGA and GSE31210 database is listed in Supplementary Table S1.

### Processing and analysis of single-cell RNA sequencing (scRNA-seq) data

The available cell clustering and cell type annotation information of GSE127465 was used in the Tumor Immune Single-cell Hub (TISCH) database (http://tisch.comp-genomics.org/) (Sun et al., 2021), and single-cell analysis was performed using the R package ‘Seurat’ (Hao et al., 2021). Based on the annotation results, M2 macrophages were extracted for further analysis. In this study, the number of hypervariable genes was set to 2000, and the resolution for cell clustering to 0.6. Principal component analysis (PCA) was conducted based on 2000 hypervariable genes. In addition, dimensionality reduction of single-cell data was used by the t-distributed stochastic neighbor embedding (tSNE) method (Kobak and Berens, 2019), and the ‘FindAllMarkers’ algorithm was performed to search for characteristic differentially expressed genes among different cell clusters. R package ‘monocle2’ was used for differentiation trajectory and pseudotime analysis of M2 macrophages (Zhou et al., 2022). Subsequently, the ‘BEAM’ (branched expression analysis modeling) statistical algorithm was used to identify heterogeneous genes that play a key role in the differentiation of M2 macrophages (Wang et al., 2022). R package ‘GSVA’ and ‘scMetabolism’ determined the enrichment of signaling pathways at the single-cell level (Hanzelmann et al., 2013; Wu et al., 2022). Finally, cell-to-cell communication analysis was performed using the R package ‘iTALK’ (Wang Y. et al., 2019).

### Functional enrichment analysis

Gene Ontology (GO) and Kyoto Encyclopedia of Genes and Genomes (KEGG) enrichment assays were performed using the R package ‘clusterprofiler’ (Wu T. et al., 2021; Kanehisa et al., 2021). GO analyses include three parts: biological process (BP), cell composition (CC), and molecular function (MF). In addition, the R package ‘limma’ was used to identify differentially expressed genes in the prognostic signature between high- and low-risk groups (Ritchie et al., 2015). Gene Set Variation Analysis (GSVA), an unsupervised algorithm, was performed to calculate enrichment scores of hallmark gene sets (Molecular Signatures Database (MSigDB), http://www.gsea-msigdb.org/gsea/msigdb/collections.jsp).

### Construction of prognostic signature

Univariate Cox analysis of heterogeneous genes associated with M2 macrophage differentiation was performed using the R package ‘survival’ to screen for prognostic genes associated with lung adenocarcinoma among them. In addition, the TCGA datasets were randomly grouped on a 3: 2 scale by the “sample_frac” function in the R package “dplyr” to obtain the training and testing datasets. Based on these prognostic genes, a Lasso-Cox regression analysis was performed using the R package ‘glmnet’ (Friedman et al., 2010; Wang H. et al., 2019). Next, a multivariate Cox stepwise regression approach was performed to construct a prognostic model related to the differentiation trajectory of M2 macrophages. The formula for the signature was: risk score = [Coef (gene 1) x gene Exp (1)] + [Coef (gene 2) x gene Exp (2)] +. . . . . . + [Coef (gene 1) x gene Exp (i)]. R packages ‘survival’ and ‘survminer’ were used for Kaplan-Meier prognostic analysis. The R package ‘timeROC’ was used to assess AUC values for time-dependent ROC curves. To further improve the prediction efficiency of the risk score, the R package ‘rms’ was used to combine the pathological stage with the risk score to construct a more accurate nomogram (Balachandran et al., 2015).

### Immune infiltration analyses of prognostic signature

Estimate, EPIC, MCPcounter, TIMER, and ssGSEA algorithms were used to calculate immune infiltration abundance in lung adenocarcinoma patients with different risk scores. Among these five algorithms, the Estimate algorithm calculated the Estimate score, tumor purity, immune score, and stromal score (Yoshihara et al., 2013). The EPIC algorithm calculated the abundance of seven immune cell types (Racle et al., 2017). The MCPcounter algorithm calculated the abundance of 10 immune cell types (Becht et al., 2016). The TIMER algorithm calculated the abundance of six immune cell types (Li et al., 2020). Subsequently, the ssGSEA algorithm was utilized to calculate the enrichment score of 24 immune cell gene sets (Bindea et al., 2013). These algorithms revealed differences in immune cell infiltration abundance between high- and low-risk groups.

### Mutation analysis, and prediction of immunotherapeutic and chemotherapy responses

Based on the TCGA mutation data (maf format), mutations in the high- and low-risk groups were analyzed using the R package ‘Maftools’ and mapped waterfall plots (Mayakonda et al., 2018). In addition, the TIDE algorithm was utilized to analyze the sensitivity of high- and low-risk groups to immune checkpoint inhibitors (ICIs) (Jiang et al., 2018). Based on the signature formula constructed above, the risk score of lung adenocarcinoma patients was calculated in GSE126044 to assess differences in immunotherapy efficacy. Subsequently, the chemotherapeutic drug sensitivity (IC50) of patients in the high- and low-risk groups was analyzed using the R package ‘pRRophetic’ (Geeleher et al., 2014). These studies helped provide personalized treatment strategies.

### Statistical analysis

Statistical analyses were performed using R software (v 4.1.3), and the results were visualized using the R packages. For non-normally distributed data, Wilcoxon rank-sum test, as a non-parametric test method, was used to examine the differences between the two groups of continuous variables, while for three and more groups we used the Kruskal–Wallis test for statistical testing. Using the Cox regression method, Kaplan-Meier prognostic analysis calculated the hazard ratio (HR). A two-sided *p* < 0.05 was considered statistically significant. Spearman method was applied for correlation analysis (*p < 0.05, **p < 0.01).

## Results

### scRNA-seq and cell annotation of lung adenocarcinoma samples

**FIGURE 1:**
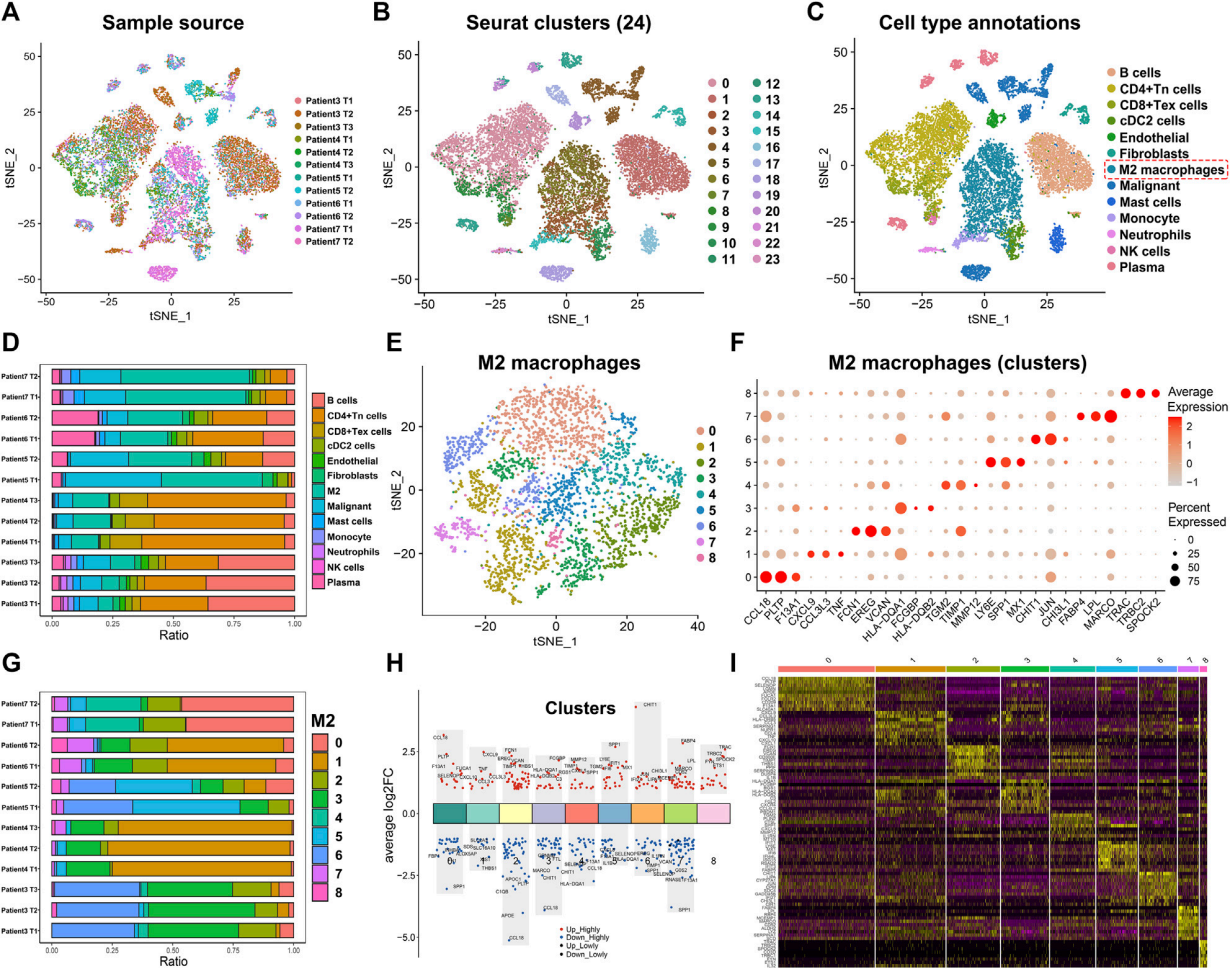
Annotation of single-cell data and extraction of M2 macrophage. **(A)** The tSNE map shows the distribution of 12 lung adenocarcinoma samples from the single-cell database GSE127465. **(B)** The tSNE plot shows that all the cells in the 12 lung adenocarcinoma samples can be classified into 24 clusters. **(C)** The tSNE map indicating that lung adenocarcinoma samples can be annotated as 13 cell types in the tumor microenvironment (different colors represent different cell types). **(D)** The histogram showing the proportion of 13 cell types in each of the 12 samples (“Patient7 T2” indicates the second tissue of patient7). **(E)** M2 macrophages classified into 9 clusters. **(F)** The bubble chart highlights the characteristic genes of different clusters **(G)** The bar chart shows the proportion of 9 clusters in each lung adenocarcinoma sample (“Patient7 T2” indicates the second tissue of patient7). **(H,I)** Biomarker genes in the 9 clusters of M2 macrophages.

To better understand the heterogeneity of M2 macrophages in the TME of lung adenocarcinoma and its potential value for prognosis and drug treatment screening, we extracted and analyzed lung adenocarcinoma samples at the single-cell level. Based on the meta-information and cell type annotation information from the GSE127465 database on the TISCH website, we extracted 12 lung adenocarcinoma samples that had been quality-controlled and standardized. To overcome technical noise in scRNA-seq data, we performed Principal Component Analysis (PCA), and each principal component (PC) was considered a “meta-feature” (Supplementary Figure S1A). We identified the most suitable number of PCs (24 PCs) for downstream analysis by calculating the standard deviation of each principal component (Supplementary Figure S1B). In addition, we used tSNE, a nonlinear dimensionality reduction algorithm, to demonstrate the distribution of single-cell data from 12 lung adenocarcinoma samples (Figure 1A). We also examined the cell distribution of lung adenocarcinoma patients of different ages and clinical stages (Supplementary Figure S1C, D). Subsequently, we used the R package ‘Seurat’ to classify the cells in 12 samples into 24 clusters (Figure 1B). These 24 clusters can also be categorized into 13 cell types: B cells, CD4^+^ Tn cells, CD8^+^ Tex cells, endothelial cells, fibroblasts, M2 macrophages, malignant cells, mast cells, monocyte cells, neutrophils cells, NK cells, and plasma cells (Figure 1C). We counted the frequency of these 13 types of immune cells and found a higher proportion of M2 macrophages in each of the 12 samples (Figure 1D). We then extracted the M2 macrophages and re-clustered them using the ‘Seurat’ package (Figure 1E). The result suggested that M2 macrophages can be classified into 9 clusters (0–8) based on different molecular markers (Figure 1F). M2 macrophages of lung adenocarcinoma patients showed considerable heterogeneity in the different clusters. Patient seven had a more significant proportion of cluster0 and cluster4 in the M2 macrophages (Figure 1G). Patient six and patient four had a high percentage of cluster1. Patient five had a larger ratio of cluster5 and cluster6. However, patient three had a greater portion of cluster3 and cluster6. Based on the above clustering results, we analyzed the signature genes of the 9 clusters of M2 macrophages using the ‘FindAllMarkers’ algorithm and visualized the results with scatter plots and heat maps (Figures 1H,I). We observed that the genes had distinct expression differences between different clusters.

### Differentiation trajectory of M2 macrophages in tumor immune microenvironment

**FIGURE 2:**
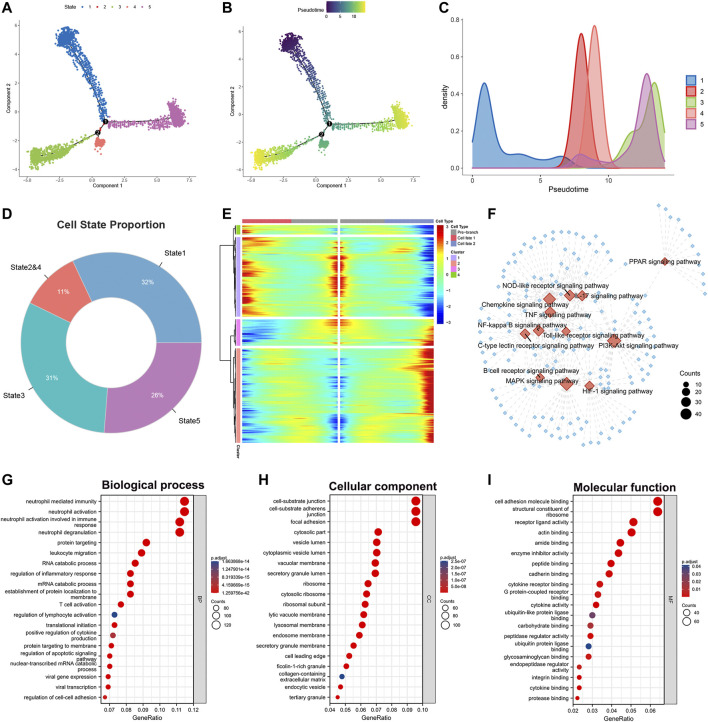
Identification of heterogeneous genes associated with differentiation trajectories of M2 macrophages. **(A)** Differentiation trajectory analysis of M2 macrophages. **(B,C)** Pseudotime analysis of M2 macrophages. **(D)** The proportion of each state in the differentiation trajectory of M2 macrophages. **(E)** The heat map revealed that M2 macrophages could exhibit four expression patterns after differentiation. **(F)** KEGG enrichment analysis of heterogeneous genes associated with differentiation of M2 macrophages. **(G–I)** GO enrichment analysis of heterogeneous genes associated with M2 macrophage differentiation.

The heterogeneity among M2 macrophages was intriguing, and to further investigate the biological functions of essential genes in the differentiation of M2 macrophages, we performed differentiation trajectory analyses. We found that M2 macrophages can be divided into five differentiation states (Figure 2A). Meanwhile, we found that subpopulations of M2-type macrophages were differentially distributed on differentiation trajectory (Supplementary Figure S1E). Subsequently, we performed pseudotime analysis on M2 macrophages (Figures 2B,C). Purple indicated the initial state of cell differentiation, and yellow indicated the terminal state. Since state2 had a smaller number of cells and a high overlap with state4 in differentiation trajectory and pseudotime, we merged state2 with state4 as a whole. State1 accounted for 32%, state2&4 accounted for 11%, state3 accounted for 31%, and state5 accounted for 26% of all M2 macrophages (Figure 2D). In addition, we found that heterogeneous genes associated with the differentiation trajectory of M2 macrophages illustrated four expression patterns (Figure 2E).

We performed KEGG and GO analysis based on these statistically significant heterogeneous genes (*p* < 0.0001). KEGG enrichment analysis indicated that these heterogeneous genes were involved in activating numerous signaling pathways (Figure 2F). Examples include the Chemokine, IL-17, HIF-1, B cell receptor, and PI3K-Akt signaling pathway. The diverse activation levels of these pathways suggested that the different states of M2 macrophages might play distinct roles in the progression of lung adenocarcinoma. GO analysis phenotyped the heterogeneous genes in biological processes, cellular components, and molecular functions (Figures 2G–I, Supplementary Table S2). The results revealed that these genes activate multiple immune cells in the TME, suggesting crosstalk between M2 macrophages and immune cells.

### Differential states of M2 macrophages reveal the heterogeneity of function characteristics and cellular communication levels

**FIGURE 3:**
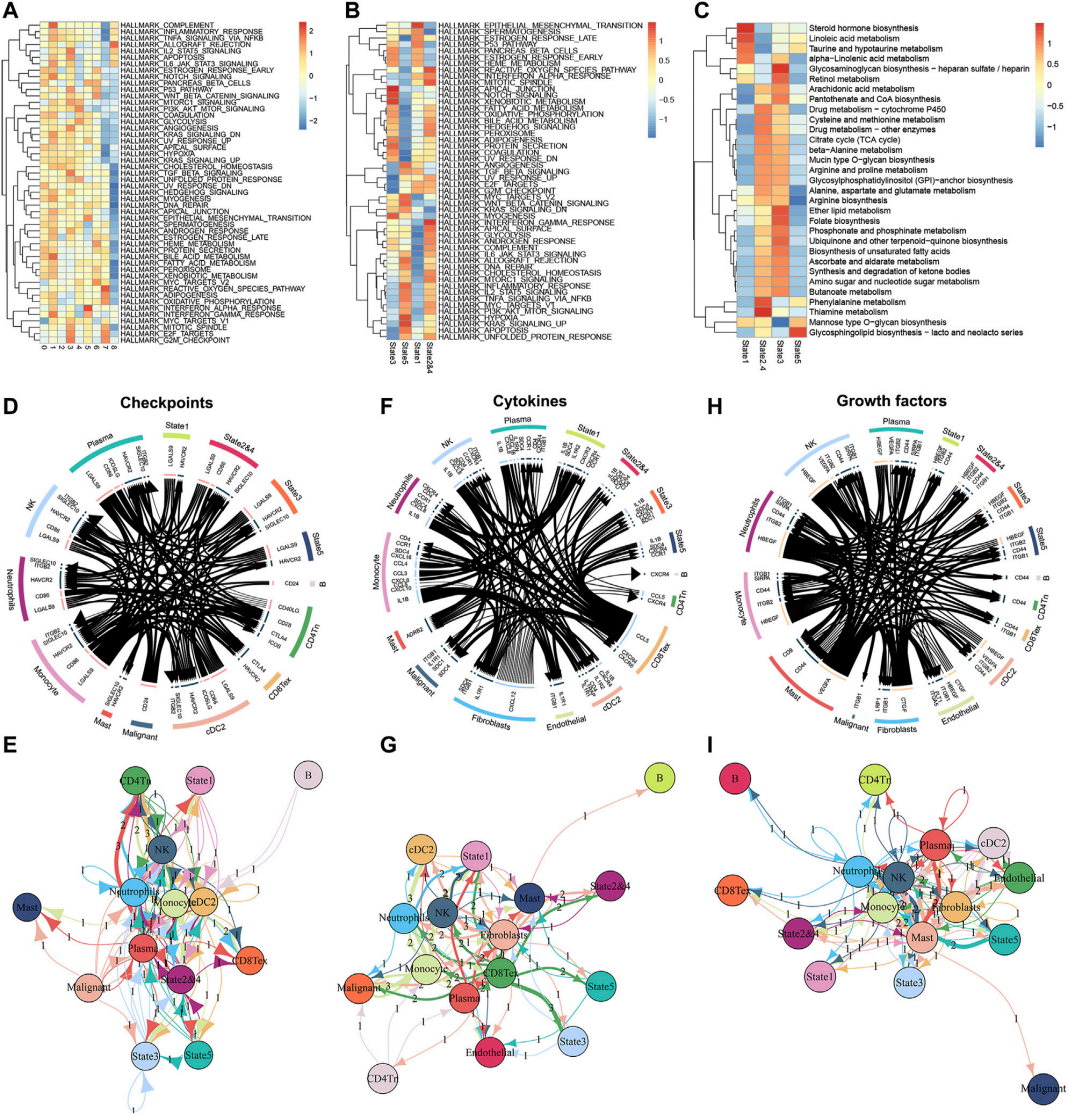
Enrichment analyses of M2 macrophages in different differentiation states and cell communication analyses in the TME. **(A)** The GSVA algorithm demonstrates different enrichment levels of 9 clusters (M2 macrophages) in the Hallmark pathway. **(B)** The GSVA analysis of differentiation states of M2 macrophages. **(C)** Enrichment scores of metabolism-related pathways in different differentiation states of M2 macrophages. **(D,E)** Communication between M2 macrophages of different differentiation states and various cell types in the tumor microenvironment at the level of checkpoints. **(F,G)** Communication between M2 macrophages of different differentiation states and various cell types in the tumor microenvironment at the level of cytokines. **(H,I)** Communication between M2 macrophages of different differentiation states and various cell types in the tumor microenvironment at the level of growth factors.

To further examine the functional differences between the different classes of M2 macrophages we distinguished, we performed a GSVA enrichment analysis. First, we verified the potential differences in molecular mechanisms among the 9 clusters of M2 macrophages. Although M2 macrophages exhibited pro-oncogenic activity, the different clusters of M2 macrophages showed significant differences in the activation levels of the 50 gene sets contained in Hallmark (Figure 3A). For instance, cluster7 and cluster8 have lower activation levels in numerous pathways than the other seven types of clusters. This suggested that cluster7 and cluster8 might be relatively weak in oncogenic activities. To better understand this heterogeneity within M2 macrophages, we also performed an enrichment analysis of the different states in the differentiation trajectory (Figure 3B). The results confirmed substantial heterogeneity among the different states. It was found that state1 had significantly higher enrichment levels in the epithelial-mesenchymal transition (EMT). In comparison, states2&4 had significantly higher activation levels in the reactive oxygen species (ROS) pathway, mitotic spindle, and interferon-gamma response. State3 was significantly activated on the apical junction and notch signaling and had the lowest activation on E2F targets, G2M checkpoint, and the genes upregulated by ultraviolet (UV) radiation. State5 was notably enriched in angiogenesis, hypoxia, and genes upregulated by KRAS signaling. Subsequently, we analyzed the differences in the metabolic activities of M2 macrophages in these states using the R package ‘scMetabolism’ (Figure 3C). The red color in the heat map corresponds to the higher activation level, and we can identify that the different states of M2 macrophages have distinct metabolic levels. This heterogeneity in metabolic levels may reveal differences in the functional levels of the M2 macrophages in different states. Finally, we analyzed the cellular communication between M2 macrophages and the other cells in the TME (immune checkpoints, cytokines, and growth factors). Regarding immune checkpoints (Figures 3D,E), we found that the M2 macrophages of state1 mainly communicated with NK cells, neutrophils, monocyte cells, and cDC2 cells. M2 macrophages in state2&4 communicated with malignant cells, CD4^+^ Tn cells, and CD8^+^ Tex cells in addition to the above 4 cells. M2 macrophages in state3 had extensive communication with plasma cells and malignant cells. In contrast, M2 macrophages in state5 communicated predominantly with monocyte cells. In terms of cytokines, the different states of M2 macrophages also have diverse levels of cellular communication (Figures 3F,G). However, in terms of cell growth factors, there was no significant difference in the level of communication between these states of M2 macrophages and immune cells (Figures 3H,I). Together, the above results demonstrated heterogeneity in the level of cellular communication among different states of M2 macrophages.

### Prognostic signature based on heterogeneous genes associated with differentiation of M2 macrophages can accurately predict lung adenocarcinoma patients’ outcome

**FIGURE 4:**
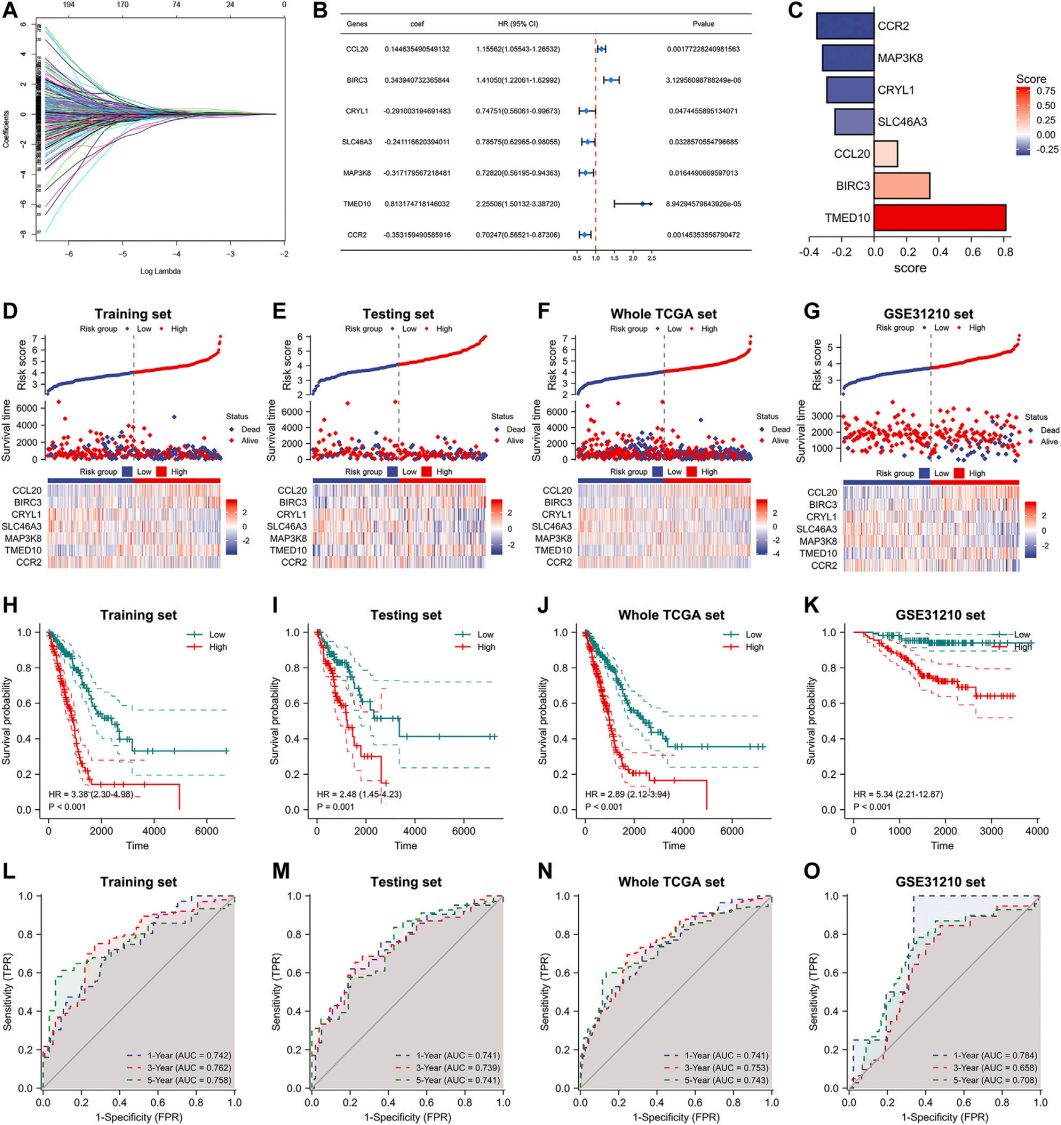
Construction of M2 macrophage differentiation-related prognostic signature. **(A)** Lasso Cox analysis of prognostic genes associated with M2 macrophage differentiation. **(B)** Multifactorial stepwise regression to construct a 7-gene prognostic model. **(C)** Coefficients of seven genes in the model formula. **(D–G)** Risk factor diagrams of signatures in the training, testing, whole TCGA, and GSE31210 dataset. **(H–K)** Kaplan-Meier prognostic analysis of signatures in the training, testing, whole TCGA, and GSE31210 dataset. **(L–O)** Time-dependent ROC curves of signatures in the training, testing, whole TCGA, and GSE31210 dataset.

After extracting heterogeneous genes associated with the differentiation trajectory of M2 macrophages, we performed a univariate Cox analysis in the TCGA database and obtained 289 prognosis-related genes of lung adenocarcinoma. Utilizing Lasso-Cox with multivariate stepwise regression, we constructed a prognostic model for lung adenocarcinoma in the training set (Figures 4A,B). In addition, Figure 4C shows the coefficients of the seven genes incorporated into the formula. These seven genes were: CCL20, BIRC3, CRYL1, SLC46A3, MAP3K8, TMED10, and CCR2. The formula of the model was: risk score = [0.144635490549132 * CCL20 Exp] + [0.343940732365844 * BIRC3 Exp] + [-0.291003194691483 * CRYL1 Exp] + [-0.241116620394011 * SLC46A3 Exp] + [-0.317179567218481 * MAP3K8 Exp] + [0.813174718146032 * TMED10 Exp] + [-0.353159490585916 * CCR2 Exp]. Using this formula, we calculated the risk score values in the testing set, the entire TCGA database, and the external validation dataset GSE31210. Lung adenocarcinoma patients with high-risk scores had higher deaths in these four datasets (Figures 4D–G). The heat map results also indicated that the expression of the above seven genes had significant differences in the two risk groups. We then performed Kaplan-Meier prognostic analysis to explore the potential value of our constructed model for patients with lung adenocarcinoma. Patients with high-risk scores had a worse prognosis in the training set (HR = 3.38, *p* < 0.001), the testing set (HR = 2.48, *p* = 0.001), the entire TCGA set (HR = 2.89, *p* < 0.001), and the GSE31210 database (HR = 5.34, *p* < 0.001, Figures 4H–K). We also performed a time-dependent ROC curve analysis on these four databases to judge our model’s accuracy in predicting prognosis. The AUC values of our model in the training set for 1-, 3-, and 5-years overall survivals were 0.742, 0.762, and 0.758, respectively (Figure 4L–O). In the testing set, the AUC values of our model for 1-, 3-, and 5-years survival were 0.741, 0.739, and 0.741, respectively. In the entire TCGA dataset, the AUC values of our model for 1-, 3-, and 5-years survival were 0.741, 0.753, and 0.741, respectively. While, in the GSE31210 dataset, the AUC values of our model for 1-, 3-, and 5-years survival were 0.784, 0.658, and 0.708, respectively.

**FIGURE 5:**
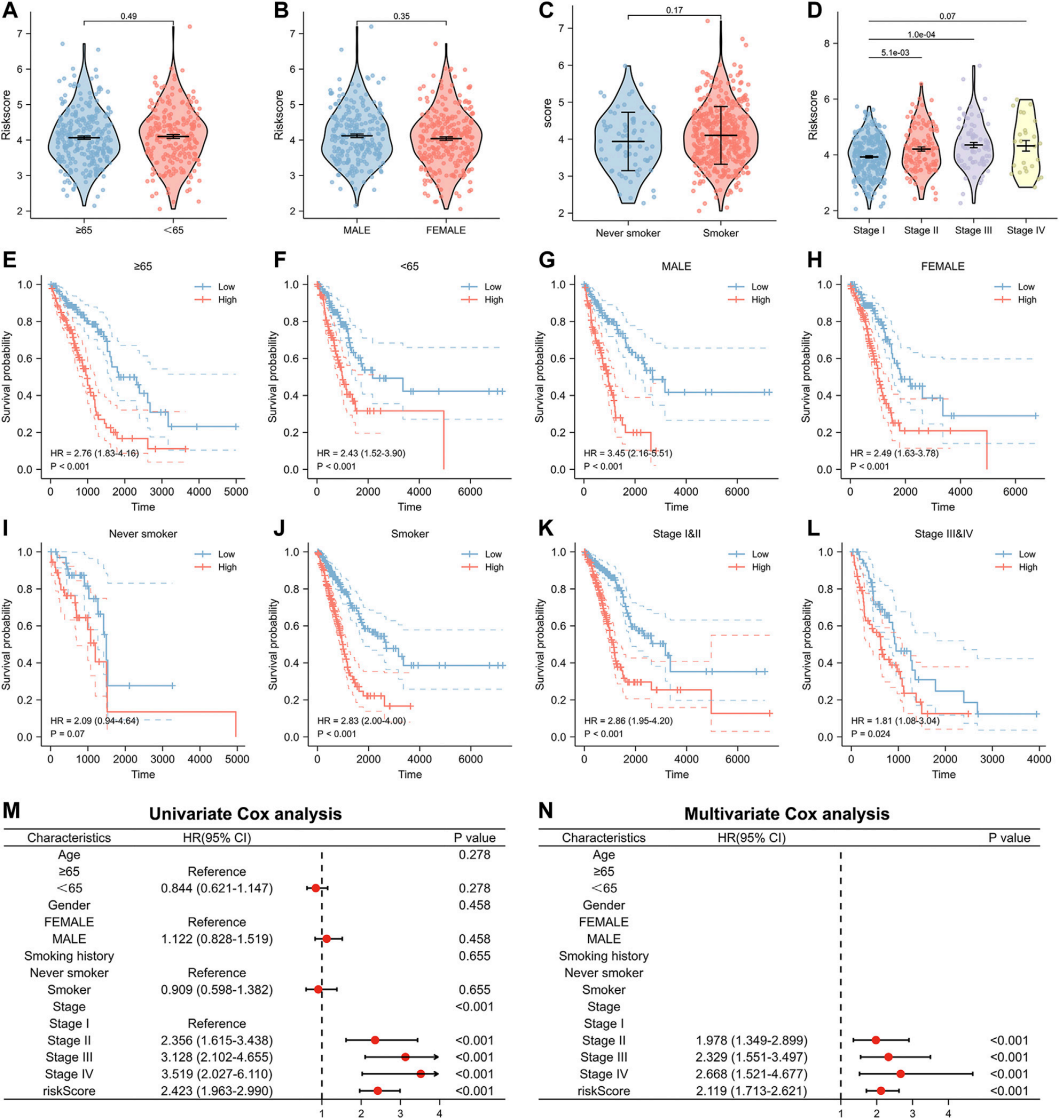
Relationship between risk score and clinical factors in the model. **(A–D)** The risk score of patients with different clinical factors. **(E,F)** Relationship between risk score and prognosis in different age groups. **(G,H)** Relationship between risk score and prognosis in different gender groups. **(I,J)** Relationship between risk score and prognosis in different smoking history groups. **(K,L)** Relationship between risk score and prognosis in different pathological stage groups. **(M)** Univariate and **(N)** multivariate Cox analysis of risk score and clinical factors.

Furthermore, we analyzed the relationship between numerous clinical factors and risk scores (Figures 5A–D). It was found that there was no statistical difference in the risk score between the two groups of patients aged≥65 years and those aged<65 years. The difference between the two risk groups of patients with different gender and smoking history was also not statistically different. However, the differences among patients with different pathologic stages were statistically significant. Patients with high pathological stages tended to have higher risk scores. In addition, we performed Kaplan-Meier prognostic analysis of lung adenocarcinoma patients with different clinical characteristics separately (Figure 5E–L). The results showed that patients with high-risk scores had a poor prognosis in all age groups (≥65, <65), all gender groups (Male, Female), and all pathological stage groups (Pathological stage IandII, Pathological stage IIIandIV). There was a significant prognostic difference between the high- and low-risk groups among the smoking group, with patients in the high-risk group having a poor prognosis (HR = 2.83, *p* < 0.001), however, there was no significant prognostic difference between the high- and low-risk groups in the non-smoking group. We also performed the univariate and multivariate Cox regression analyses regarding the risk scores. Our results showed that the risk score is an independent prognostic factor for lung adenocarcinoma and can be used as a clinical parameter to determine the prognosis of patients (Figure 5M, N).

### Significant difference in molecular mechanisms and immune infiltration levels between high- and low-risk groups

**FIGURE 6:**
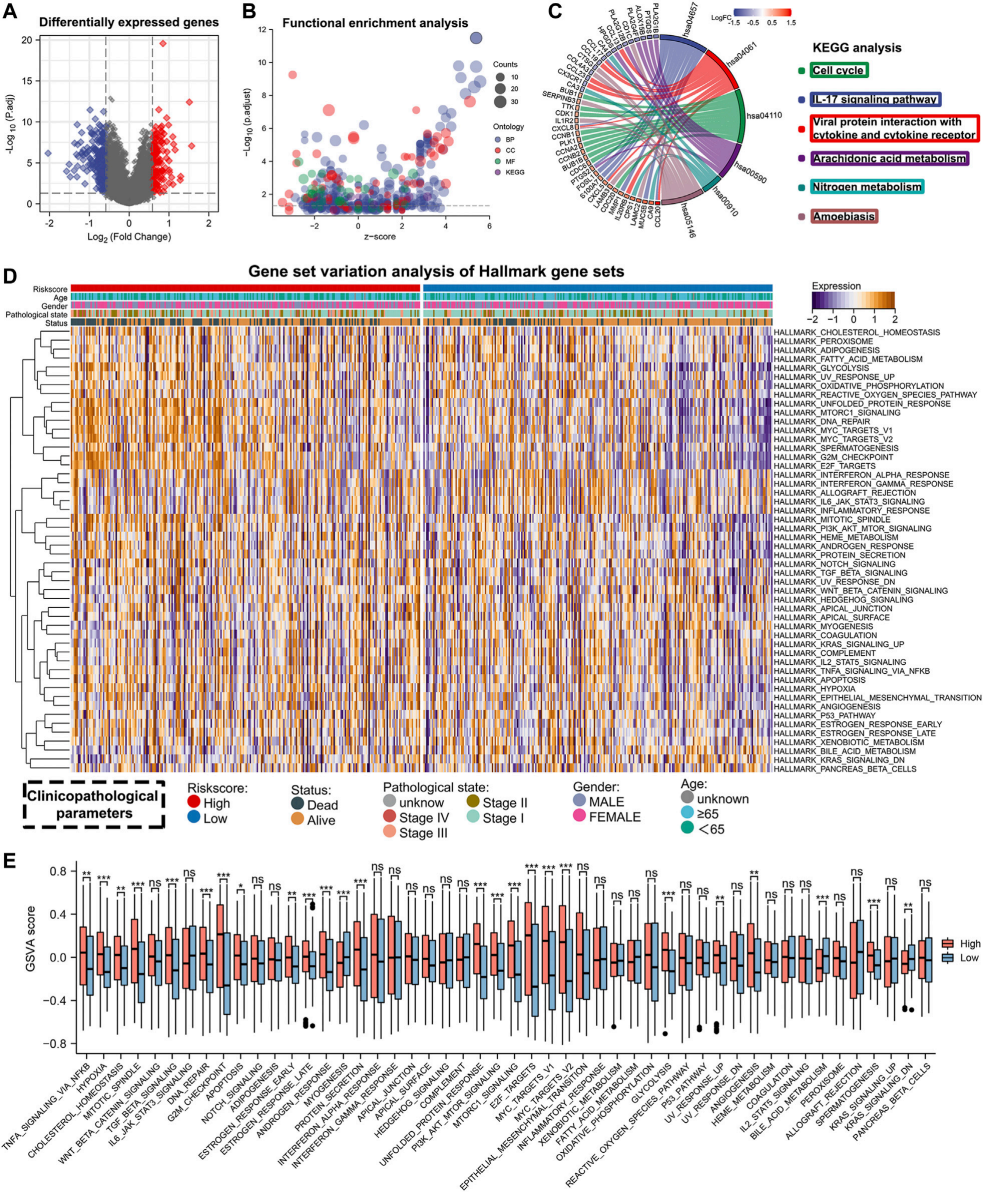
Enrichment analysis of differentially expressed genes between high- and low-risk groups. **(A)** Differentially expressed genes between high- and low-risk groups (FDR <0.05, **|**FC**|** > 1.5). **(B)** GO and KEGG enrichment analysis for differentially expressed genes. **(C)** Visualization of KEGG enrichment analysis results. **(D,E)** The heat map and bar chart shows the GSVA analysis results between the high- and low-risk groups.

As demonstrated in the above study, lung adenocarcinoma patients had significantly different prognoses between the high- and low-risk groups. Differential expression analysis was performed for the high-risk versus low-risk groups to investigate the mechanisms involved. Firstly, we used the R package ‘limma’ to identify differentially expressed genes (**|**FC**|**>1.5, FDR<0.05) and mapped the volcano (Figure 6A), Then, we performed enrichment analysis using GO and KEGG (Figure 6B, Supplementary Table S3). KEGG results showed significant enrichment in the cell cycle and IL-17 signaling pathway (Figure 6C). In addition, we performed GSVA enrichment analysis and plotted heat map and histogram for lung adenocarcinoma patients in high- and low-risk groups. Fifty gene sets from Hallmark were selected for GSVA analysis. By comparing the enrichment scores of the two groups in these 50 gene sets, 25 gene sets showed a statistically significant difference (Figures 6D,E). These results might explain the underlying mechanism for the difference in the prognosis of lung adenocarcinoma patients with different risk scores.

**FIGURE 7:**
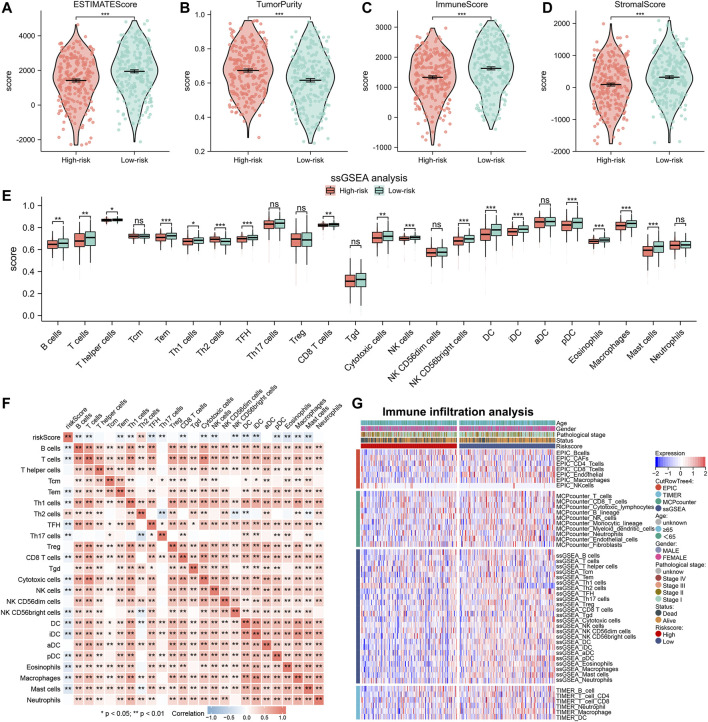
Immune infiltration analysis between high- and low-risk groups. (**A–D**) Immune infiltration abundance between high- and low-risk groups by Estimate algorithm. **(E)** The histogram displays differences in infiltrating abundance of 24 immune cell types in high- and low-risk groups analyzed by the ssGSEA algorithm. **(F)** Correlation between the risk score and 24 immune cell types. **(G)** The heat map showing the immune infiltration analysis results of EPIC, TIMER, MCPcounter, and ssGSEA.

To understand the differences in TME among patients with different risk scores, we also analyzed the abundance of immune infiltration in high- and low-risk groups. Analysis using the Estimate algorithm showed that the high-risk group had higher tumor purity but lower immune and stromal scores (Figures 7A–D). This suggested that patients with high-risk scores exhibited a state that promoted tumor escape due to the lack of anti-tumor immune cells in the TME. Then, to investigate the immune status of the two risk groups in more detail, we used the ssGSEA algorithm to calculate the infiltration abundance of 24 immune cell types (Figure 7E). The results revealed that the high-risk group generally had a lower infiltration abundance of immune cells, including B cells, T cells, CD8^+^ T cells, NK cells, DC cells, and mast cells, compared to the low-risk group. Spearman correlation analysis also showed that the abundance of immune cells was negatively correlated with the risk scores for almost all immune cell types except Th2 cells (Figure 7F). We also used the MCPcounter, TIMER, and EPIC algorithms to confirm these results. By plotting the heat map, we visualized that lung adenocarcinoma patients in the high-risk group had lower levels of immune infiltration (Figure 7G).

### Risk score can suggest disparities in gene mutations and guide immunotherapy and chemotherapy

**FIGURE 8:**
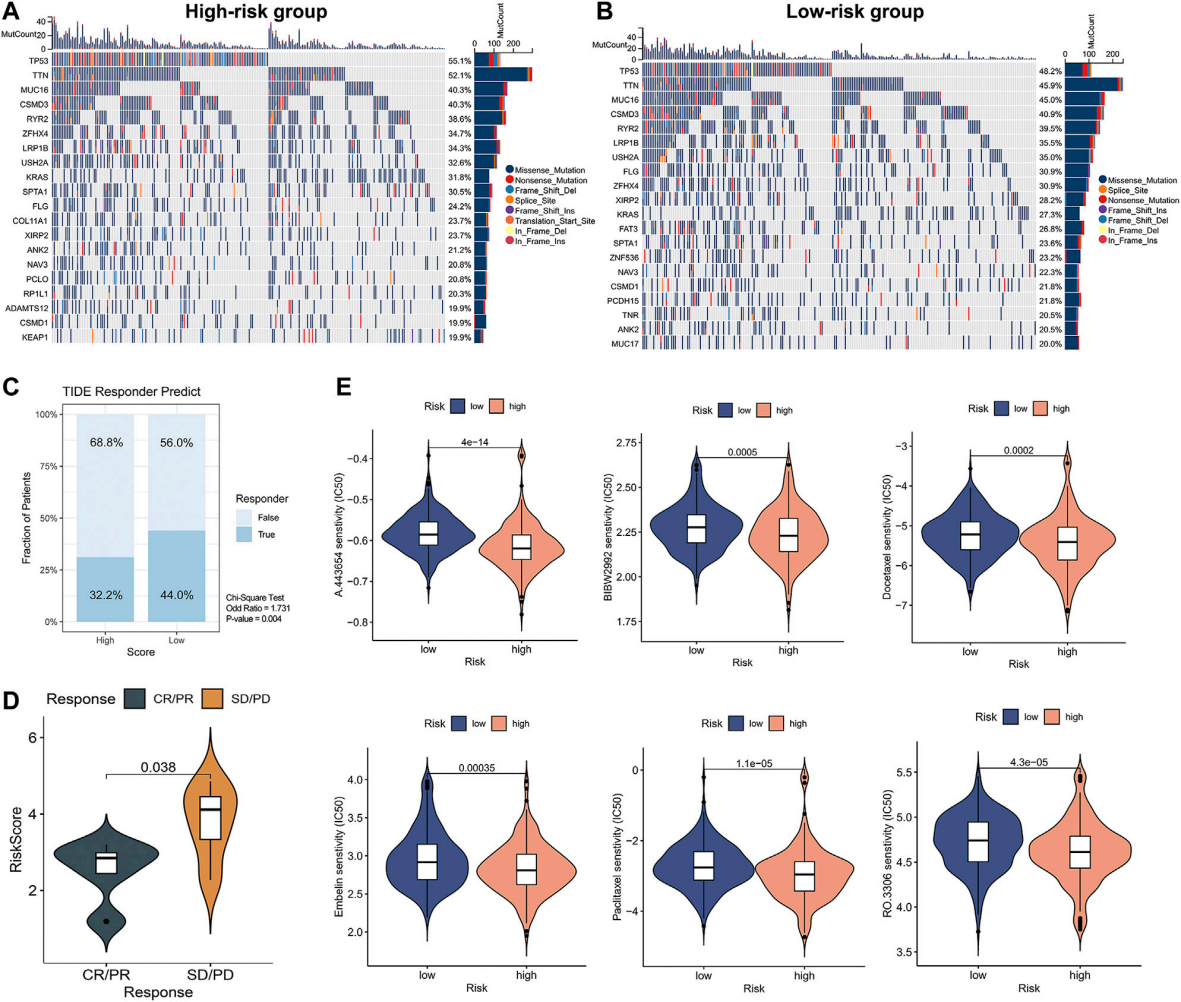
Mutation status and prediction of immunotherapy and chemotherapeutic response between high- and low-risk groups. **(A,B)** Analysis of top 20 mutation genes in high-risk versus low-risk groups. **(C)** TIDE immunotherapy response prediction in high- and low-risk groups of LUAD TCGA cohort. Chi-square test: no response to immunotherapy: high-versus low-risk, 68.8% versus 56.0%, OR = 1.731, *p* = 0.004. **(D)** The Independent immunotherapy dataset (GSE126044) validated immunotherapy efficacy in high- and low-risk groups. **(E)** Screening of chemotherapeutic agents sensitive to high- and low-risk groups.

Since gene mutation status significantly affects tumor formation and progression, we performed a mutation analysis of lung adenocarcinoma patients in high- and low-risk groups. We further analyzed the differences between the top 20 genes with the highest mutation frequencies in the high and low-risk groups respectively (Figures 8A,B). The frequency of TP53 mutations in the high-risk group was 55.1%, while it was 48.2% in the low-risk group. The frequency of KRAS mutations was 31.8% in the high-risk group versus 27.3% in the low-risk group. The difference in gene mutation frequency might be the reason for the poorer prognosis in the high-risk group. Based on the above analyses, we explored the treatment strategies for different risk scores in depth. Since immunotherapy is commonly used in lung adenocarcinoma, we first calculated the response to immunotherapy in high- and low-risk groups using the TIDE algorithm (Figure 8C). The results suggested that the low-risk group had a better treatment response upon immunotherapy (OR = 1.731, *p* = 0.004). We further validated this result using the GSE126044 database. Results showed that patients with CR/PR after immunotherapy had a significantly lower risk score than SD/PD (*p* = 0.038), suggesting that patients with a low-risk score are susceptible to benefit from immunotherapy (anti-PD-1 treatment) (Figure 8D). However, the high-risk group had a worse prognosis, so we performed a prediction of response to chemotherapeutic agents for patients in the high- and low-risk groups. We utilized the R package ‘pRRophetic’ to calculate the IC_50_ of chemotherapeutic drugs (Figure 8E). We found that the high-risk group had better sensitivity to A-443654, BIBW-2992, Docetaxel, Paclitaxel, Embelin, and RO-3306. Taken together, we provided a personalized treatment option for clinical reference by predicting the treatment effect of patients in different subgroups and compensated for the poorer effect of immunotherapy in the high-risk group.

### A nomogram with a potential clinical application can be constructed based on risk score and pathological stage

**FIGURE 9:**
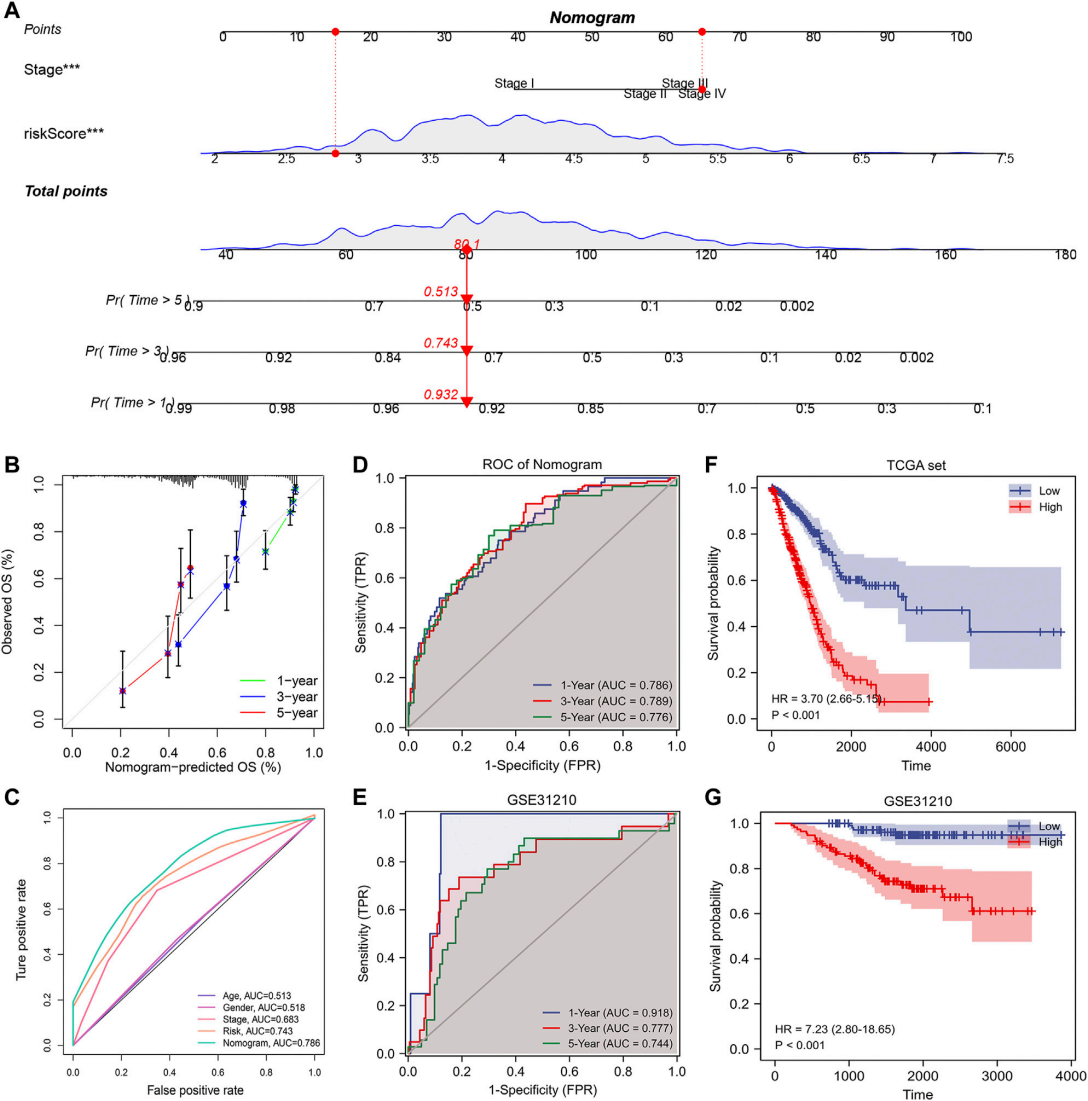
Building a more accurate nomogram. **(A)** Nomogram was constructed by combining pathological stages with risk scores. **(B)** Nomogram’s 1-, 3-, and 5-years calibration curve. **(C)** The ROC curve shows AUC values for various clinical factors, risk scores, and nomogram scores. **(D)** ROC analysis of nomogram score in TCGA database. **(E)** ROC analysis of nomogram score in GSE31210 database (validation set). **(F)** Kaplan-Meier prognostic analysis of nomogram score in TCGA database. **(G)** Kaplan-Meier prognostic analysis of nomogram score in GSE31210 database.

The above study indicated that the risk score could act as an independent prognostic factor that can be used to determine the prognosis of patients. Therefore, to further improve our signature’s predictive efficiency, we constructed a nomogram based on the TCGA database, incorporating factors such as pathological stage and risk scores (Figure 9A). We could visualize the risk assessment by calculating the score and assessing the outcome probability for each patient. In addition, we tested the predictive efficacy of the nomogram using a calibration plot (Figure 9B). The nomogram’s 1-, 3-, and 5-years survival predictions were more accurate than the theoretical values. To further compare the predictive efficacy of the nomogram score with other clinical factors, we plotted the ROC curve. The nomogram score had the highest AUC value, and the predictive efficiency was further improved based on the risk score (Figure 9C). By performing the time-dependent ROC curve based on the nomogram score, we found that the AUC values of 1-, 3-, and 5-years overall survival for lung adenocarcinoma patients were 0.786, 0.789, and 0.776, respectively. To verify the accuracy of the nomogram, we constructed the nomogram again based on the external dataset GSE31210. Time-dependent ROC analysis showed that the AUC values of 1-, 3-, and 5-years overall survival for lung adenocarcinoma patients were 0.918, 0.777, and 0.744, respectively (Figures 9D,E). These results suggested that the nomogram we constructed had good accuracy. We also performed Kaplan-Meier prognostic analyses using the TCGA and external database GSE31210. The results indicated that the high nomogram score group had a significantly worse prognosis than the low score group (Figures 9F,G). In summary, the nomogram significantly improved the accuracy of determining the survival status of lung adenocarcinoma patients.

## Discussion

The interconnection between tumor cells, immune cells, and stromal cells in the TME substantially influences tumorigenesis and tumor progression (Anderson and Simon, 2020). The spatial interplay of immune cells and other cells in the TME determines the immune response against tumors (Petitprez et al., 2020). As immunotherapy of tumors has been intensively studied, immune checkpoint inhibitors (ICIs) against tumor immune escape are expected to be an essential strategy to improve the prognosis of lung adenocarcinoma patients (Qiao et al., 2021; Liu et al., 2022; Reda et al., 2022). However, due to the heterogeneity and complexity of the TME, patients with the same pathological stage may also exhibit different TME characteristics, resulting in different therapeutic effects upon immunotherapy (Bagaev et al., 2021). Therefore, developing a prognostic model to determine the prognosis of lung adenocarcinoma patients early and to provide targeted immunotherapeutic strategies has significant potential for clinical application.

Tumor-associated macrophages, a vital member of the TME, have been identified in two types with different functional features, the classically activated M1 macrophages and the alternative activated M2 macrophages (Cassetta and Pollard, 2020). M1 macrophages appear in the inflammatory environment and are usually induced by cytokines from Th1, whereas M2 macrophages are primarily induced by cytokines from Th2 and counteract the inflammatory response (Sedighzadeh et al., 2021). Previous studies have shown that although all M2 macrophages exhibit anti-inflammatory and immunomodulatory effects, there is still heterogeneity among M2 macrophages. M2 macrophages can be further distinguished into four subtypes. M2a macrophages are involved in tissue fibrosis, M2b macrophages are shown to promote tumor progression, M2c macrophages are exhibited to be involved in tissue remodeling, and M2d macrophages promote angiogenesis (Wang L. X. et al., 2019). A growing number of studies have shown a significant correlation between M2 macrophages and lung adenocarcinoma progression. Lung adenocarcinoma patients with a higher density of M2 macrophages tend to have a poorer prognosis (Cao et al., 2019; Guo et al., 2019; Dai et al., 2020). M2 macrophages create an environment conducive to tumor survival by releasing growth factors, chemokines, and other inflammatory mediators (Solinas et al., 2009; Lin et al., 2019). In addition, M2 macrophages can also promote tumor metastasis and invasion by promoting angiogenesis and other pathways (Jetten et al., 2014; Xie et al., 2021). To further analyze the differences in composition and function within M2 macrophages, we used bioinformatics to perform an in-depth analysis at the single cell level.

Through annotation and clustering analysis of single-cell data, we identified differences in the composition of M2 macrophages in lung adenocarcinoma patients. These differences might indirectly contribute to the discrepancy in biological processes and prognosis among patients. In addition, we performed differentiation trajectory analysis and pseudo-time analysis on M2 macrophages, identifying the different differentiation states of M2 macrophages. Lung adenocarcinoma patients exhibited four expression patterns based on the heterogeneous genes in the differentiation trajectory. The GSVA enrichment analysis helped us to understand the functional differences between different states of M2 macrophages. The GSVA results from our study above showed that M2 macrophages in state5 had the highest angiogenesis score, M2 macrophages in state1 had the highest epithelial-mesenchymal transition (EMT) score, and M2 macrophages in state2&4 had the highest interferon-response score. In contrast, M2 macrophages in state3 had the lowest G2M checkpoint score. This discrepancy reveals that macrophages in different states may differ in their cancer-promoting functions. Previous studies have also shown that M2 macrophages are significantly associated with angiogenesis and lymphangiogenesis, which contribute to the development of lung cancer, and also support that M2 cells are a strong indicator of poor prognosis in lung cancer (Hwang et al., 2020). Identifying particular metastasis-promoting or EMT-promoting subtypes of M2 macrophages also can help to explore the underlying molecular mechanisms further. Additionally, this heterogeneity of different states of M2 macrophages was also reflected in the metabolic and cellular communication levels. Our results provide a novel insight into the heterogeneity in M2 macrophages. Whereas the previous classification of M2 macrophages was based on different cytokine activation patterns (Colin et al., 2014), we distinguished different differentiation states of M2 macrophages based on single cell analysis. In addition, our study investigated the role of heterogeneous genes in the differentiation of M2 macrophages to guide the clinical therapy of lung adenocarcinoma.

We extracted heterogeneous genes, essential in the differentiation trajectory of M2 macrophages, and performed a univariate Cox analysis to screen for prognosis-related genes in lung adenocarcinoma. We constructed a prognostic model using Lasso-Cox and multivariate stepwise regression methods based on the prognosis-related genes in the training set. We measured the predictive efficacy of the model and explored the potential molecular mechanisms between high- and low-risk groups. Previous studies have shown that the status of the tumor microenvironment can be quantitatively assessed by risk scores (Chong et al., 2021). In our research, we found that patients in the high-risk group had an immunosuppressive microenvironment while the low-risk group had an immune-promoting microenvironment. Notably, the treatment of immune checkpoint inhibitors (ICIs) has become a hot topic in tumor therapy strategies. Immunotherapy targeting M2-type macrophages is emerging as a new direction for tumor therapy (Mills et al., 2016). The major molecules targeted by immunotherapy are programmed death receptor 1 (PD-1) and programmed death receptor ligand 1 (PD-L1). However, due to the complexity of the *in vivo* microenvironment, immunotherapy has an obvious shortcoming in that only a fraction of tumor patients respond to ICIs treatment (Wang et al., 2021). TIDE, as a novel computational architecture, has been considered as an alternative to single biomarkers for predicting the therapeutic effect of ICIs (Jiang et al., 2018). With the dual validation of the TIDE algorithm and GSE126044 set, we found that the low-risk group benefited more from immunotherapy, and this also directly indicated that the prognostic model we constructed could advance the personalization of immunotherapy.

As the high-risk group was shown to have a poor prognosis, we identified chemotherapeutic agents (A-443654, BIBW-2992, Docetaxel, Embelin, Paclitaxel, RO-3306) with better sensitivity for the high-risk group. A-443654 is an inhibitor of the AKT pathway that induces apoptosis and inhibits tumor growth (Luo et al., 2005). BIBW-2992 was reported to inhibit the kinase activity of EGFR mutants and suppress lung adenocarcinoma development (Li et al., 2008). Docetaxel and Embelin can induce the apoptosis of lung adenocarcinoma tumor cells (Avisetti et al., 2014; Jeong et al., 2021). Meanwhile, Paclitaxel, as first-line chemotherapy for patients who do not benefit from immunotherapy, together with RO-3306, can cause cell cycle G2/M phase arrest and lead to apoptosis in lung adenocarcinoma cells (Vassilev et al., 2006; Cui et al., 2020). The above chemotherapy drugs could compensate for the deficiency in immunotherapy efficacy in the high-risk group. In addition, to further improve the predictive performance of the prognostic model, we constructed a nomogram by combining the risk scores with the pathological stages. Nomogram has significantly better prognostic efficacy than the pathological stages and can be used as a complement to clinical factors by providing a more refined risk assessment.

In summary, for the first time, this research constructed a signature that can assess the prognosis of lung adenocarcinoma patients based on heterogeneous genes related to the differentiation trajectory of M2 macrophages. Our results provide a new research idea for the precision treatment of lung adenocarcinoma. However, our study still has some shortcomings. More in-depth studies are needed in the future to identify the potential molecular mechanisms of heterogeneous genes associated with the differentiation of M2 macrophages.

## Conclusion

M2 macrophages, as a critical component of the lung adenocarcinoma microenvironment, promote tumor progression and metastasis. In this study, we performed differentiation trajectory and pseudotime analysis using scRNA-seq data to identify different differentiation states of M2 macrophages. By exploring the heterogeneous genes associated with M2 macrophages’ differentiation, we constructed a prognostic model to predict the prognosis and adjuvant treatment effect of lung adenocarcinoma patients, which could potentially be used as a clinical parameter for clinicians’ therapy decisions in the future.

## Data Availability

The original contributions presented in the study are included in the article/Supplementary Material, further inquiries can be directed to the corresponding authors.
