# Supplementary figures and images for: Integration of single-cell and bulk RNA-seq to establish a predictive signature based on the differentiation trajectory of M2 macrophages in lung adenocarcinoma
Source: Front Genet. 2022 Sep 12;13:1010440. doi: 10.3389/fgene.2022.1010440 (PMC9510778; doi:10.3389/fgene.2022.1010440)

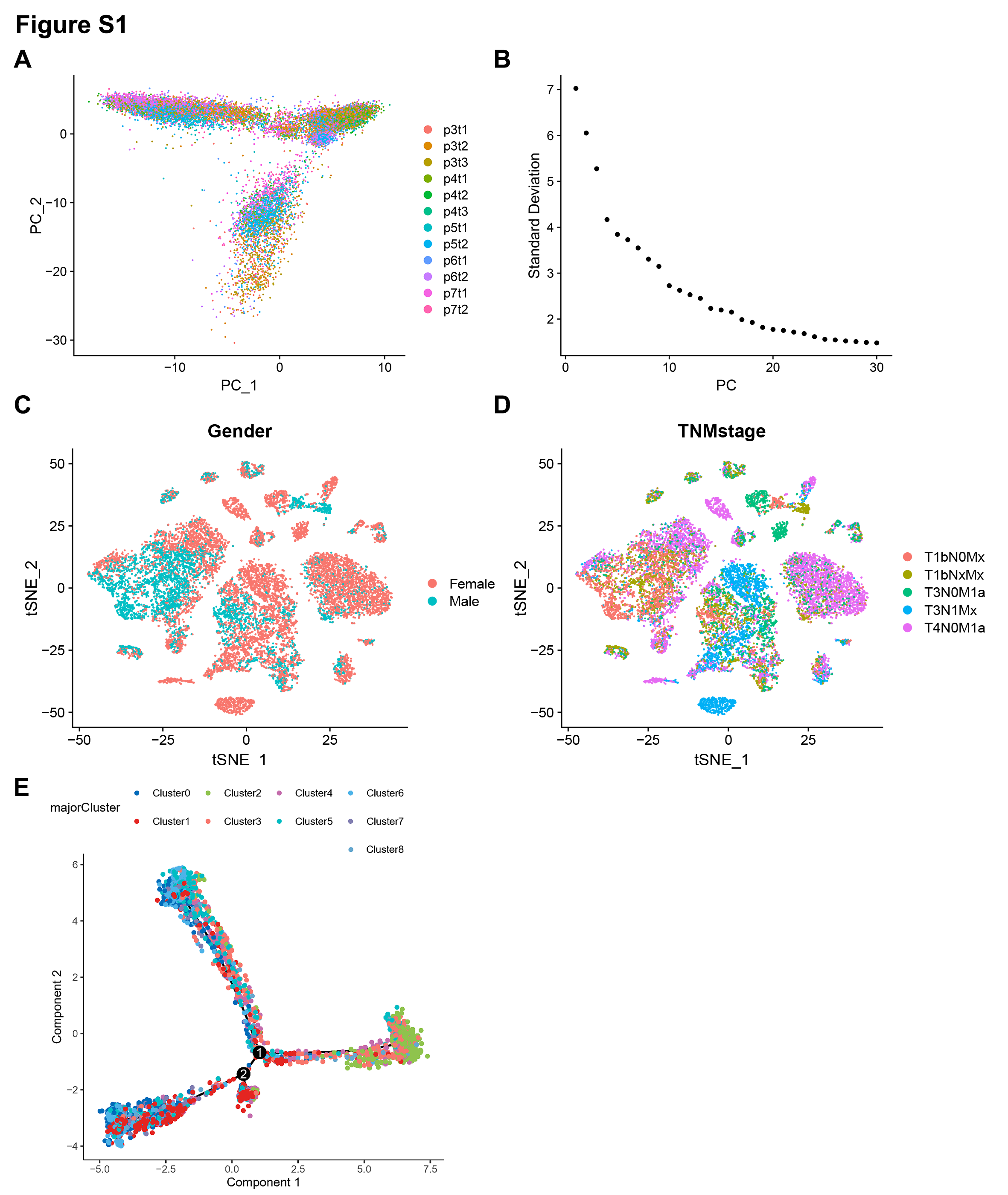

Supplement: Supplementary file 3 [file Image1.TIF]
